# The first mitogenome of the genus *Amphalius* (Siphonaptera: Ceratophyllidae) and its phylogenetic implications

**DOI:** 10.1017/S0031182024000635

**Published:** 2024-09

**Authors:** Ju Pu, Xiaoxia Lin, Wenge Dong

**Affiliations:** Yunnan Provincial Key Laboratory for Zoonosis Control and Prevention, Institute of Pathogens and Vectors, Dali University, Dali, Yunnan, China

**Keywords:** mitogenome, phylogeny, plague, Siphonaptera

## Abstract

*Amphalius spirataenius* belongs to Arthropoda, Insecta, Siphonaptera, Ceratophylloidea, Ceratophyllinae, *Amphalius*. Only 2 species from the subfamily Ceratophyllinae have been sequenced for mitogenomes to date. The genus *Amphalius* mitogenome research was still blank. The *A. spirataenius* mitogenome was determined, annotated and analysed for the first time in this study. The 14 825 bp long genome has the typical metazoan of 37 genes with insect ancestral genome arrangement pattern. There was no significant difference in codon usage of 13 protein-coding genes: UUA, UCU, GUU, ACU and GCU were the most frequently used codons. It was found that the reason for codon preference mainly contributed to natural selection base on PR2, ENC-plot and neutrality curve analysis. Evolutionary rate, conserved sites, variable sites and nucleotide diversity analysis indicated that *nad6* of *A. spirataenius* had the fastest evolutionary rate, while *cox1* had the slowest evolutionary rate. Phylogenetic trees were reconstructed based on 13 protein-coding genes and 2 rRNA genes datasets using Bayesian inference and maximum likelihood method. The phylogenetic tree supported that both Siphonaptera and Mecoptera were monophyletic, and were sister groups to each other. This study filled gap of the genus *Amphalius* mitogenome sequences and was of great significance for understanding evolution of the order Siphonaptera.

## Graphical abstract

**Figure:**
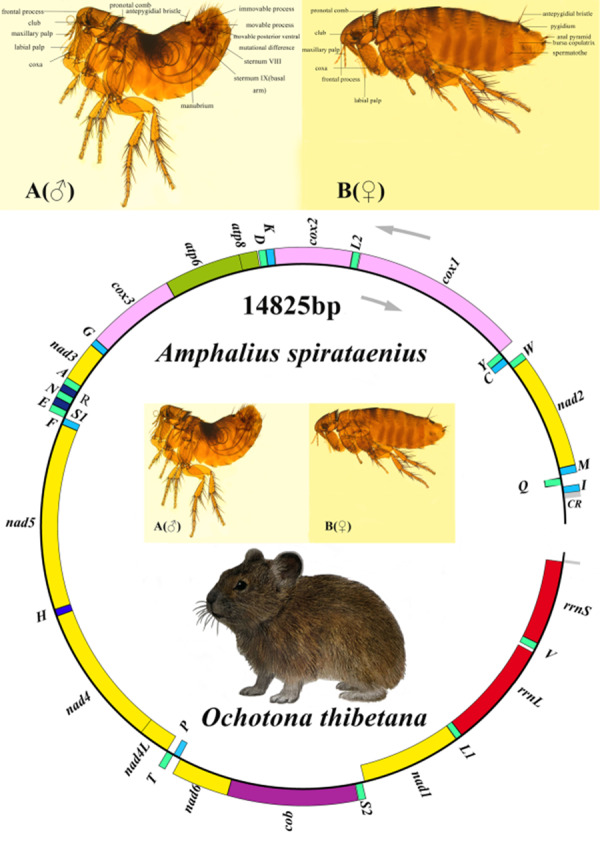

## Introduction

Fleas belong to Arthropoda, Insecta and Siphonaptera. They were important vector insects parasitized on warm-blooded animals. They mainly transmitted plague and murine typhus. Fleas undergo complete metamorphosis including egg, larva, pupa and adult stage. Larva fleas lived on organic matter in the nest and were in non-parasitic state, while adult fleas mainly parasitized on medium and small mammals (Krasnov *et al*., 2006; Eisen *et al*., 2012). As a temporary host and principal vector for plague transmission, fleas were the early warning indicator for judging plague epidemic, and also an indispensable part of plague biological community. Fleas play important role in maintaining the stability of plague natural foci (Eisen *et al*., 2012; Zhao, 2017). As the ‘accomplice’ of plague transmission, fleas have always attracted considerable attention. However, due to backward sequencing technology previously, genomics research has been considerably hindered. Most researches have focused only on morphological classification and pathogen, and the accurate identification and differentiation of related species and cryptic species was often challenged (Linardi and Santos, 2012). In the 19th century, some researchers thought that fleas were beetles according to their morphological characteristic (Crowson and Hennig, 1970). In the middle and late 20th century, it was found that fleas were closely related to Mecoptera (scorpionflies) and Diptera (flies, blackfly, mosquito etc.), together constituting the group Antliophora (Kristensen and Niels, 2009). In the 21st century, with the development of molecular systematics and genome sequencing technology, it was found that Siphonaptera and Mecoptera were sister groups (Chalwatzis *et al*., 1996; Whiting, 2004). In recent years, Tihelka *et al*. proposed to classify the order Siphonaptera into infraorder and reduce the order number of holometabolous insects to 10 orders (Tihelka *et al*., 2020); and it was thought that Siphonaptera were placed into the order Mecoptera, and the order Siphonaptera and the family Nannochoristidea were sister groups. There were about 2500 species (subspecies) of Siphonaptera in the world and 650 species (subspecies) in China (Wu, 2007). So far, only 17 flea mitogenomes in the order Siphonaptera have been sequenced. With the recent advances of high-throughput sequencing technology, more and more flea mitogenomes have been sequenced.

*Amphalius spirataenius* was first collected on *Ochotona thibetana* in 1963 (Liu *et al*., 1966). In 1975, Smit gave detailed description of the morphological characteristics of male and female individuals of *A. spirataenius* (Smit, 1975). This species was later collected in Qinghai, Yunnan, Sichuan and other places (Li, 1980; Ji *et al*., 1981; Cai, 1997). In the past, most studies on *A. spirataenius* focused on morphological structure and living habits, while the study of mitogenome was still blank. In this study, the *A. spirataenius* mitogenome was determined and analysed for the first time, and its morphological characteristics were described in detail. Combined with mitogenomes of the order Siphonaptera from GenBank for comparative analysis, and the appropriate outgroups were selected to construct a phylogenetic tree, which provided molecular biological genetic data for better promoting rapid and reliable identification of fleas.

## Materials and methods

### Specimen collection, morphological identification, DNA extraction and mitogenome sequencing

*Amphalius spirataenius* was collected from the body surface of *Ochotona thibetana* (Rodentia, Muroidea) in Deqin County, Yunnan Province, China, and species identification was done using morphological characteristics. The morphological identification was primarily based on ‘*Fauna Sinica insecta Siphonaptera*’ (Wu, 2007). Specimens were preserved in EP tubes filled with 95% ethanol and stored in a refrigerator at –80°C. Hosts and specimens were deposited in Dali University. All specimens follow small mammal capture protocol and procedures and have been approved by the Animal Ethics Committee of Dali University (No. MECDU-201912-20). Specimens were subsequently sent to Shanghai Winnerbio Technology Co., Ltd. (Shanghai, China) for DNA extraction. The integrity of the obtained total DNA was detected by gel electrophoresis. Samples with intact DNA were submitted to high-throughput sequencing on the Illumina Novoseq 6000 platform; the quality of the original data was sheared; finally, clean data were obtained.

### Mitogenome assembly, annotation and analysis

MitoZ 2.3 (https://doi.org/10.1101/489955) was used to *de novo* assemble mitogenome. The principle was that the average sequencing depth of mitogenome reads was much higher than that of nuclear genome; different Kmer parameters were set to achieve the best assembly effect. To ensure the assembly accuracy, the sequencing raw data were mapped to the assembled genome using bwa v0.7.17 (https://bio-bwa.sourceforge.net/) and samtools v0.1.20 (https://github.com/samtools/samtools/releases?page=2). The sequencing depth of the assembly result was evaluated and generally sequencing depth of 100× or higher was deemed to indicate a high level of accuracy in the assembly results. Sequence assembly was performed using Geneious Prime 11.0 (Kearse *et al*., 2012) software. The tRNA genes were predicted using tRNAscan SE (Chan *et al*., 2021) and ARWEN (Laslett and Canbäck, 2008), and protein-coding genes (PCGs) and rRNA genes were identified using Geneious Prime 11.0 software, BLAST (Altschul *et al*., 1997) and MITOS (Bernt *et al*., 2013). The annotated mitogenome sequence of *A. spirataenius* was deposited in the GenBank (accession number: OR855715) database. PhyloSuite (Zhang *et al*., 2020) was used to calculate the relative synonymous codon usage (RSCU) and nucleotide composition. Strand asymmetry was calculated: AT skew = (A − T)/(A + T) and GC skew = (G − C)/(G + C). CodonW was used to calculate the RSCU. Ka (the number of non-synonymous substitutions per non-synonymous site: Ka = dN = SA/LA), Ks (the number of synonymous substitutions per synonymous site: Ks = dS = SS/LS) and *ω* (the ratio Ka/Ks) were estimated with the software KAKS_Calculator 2.0. (Wang *et al*., 2010). Conserved sites and variable sites of 13 PCGs were calculated in DnaSP 6.0 and MEGA 11.0. Nucleotide diversity was calculated in DnaSP 6.0.

### Phylogenetic analysis

A total of 40 species were selected to construct a phylogenetic tree, including18 flea species, 21 insect species and *Philaenus spumariu*s (Stewart and Beckenbach, 2005) (NC005944) as the outgroup. Phylogenetic analysis of 13 PCGs and 2 rRNA (*rrnS* and *rnnL*) genes in 40 species was performed using maximum likelihood (ML) (Stamatakis, 2006) and Bayesian inference (BI) (Ronquist and Huelsenbeck, 2003). MAFFT (Katoh *et al*., 2002) was used for sequence alignment and MACSE (Ranwez *et al*., 2011) was used for data optimization. We estimated an ML tree using IQ-Tree and the optimal partition scheme and the best model for each partition were selected under BIC. Clade support was assessed using non-parametric bootstrap with 1000 replicates. Four independent Markov chains were run for 10 million generations in BI tree. The trees were sampled every 1000 generations with the first 25% discarded as burn-in. The phylogenetic trees were visualized and edited using FigTree 1.4.4 (http://tree.bio.ed.ac.uk/software/figtree/).

## Results

### Morphological characteristics

Eyes are larger; club segments with 9 tubercles, with frontal process and sharp frontal process, shaped like a tooth, slightly plunged into the forehead, situated slightly below the frontal margin; the lower labial palp must exceed the end of the coxa of the forefoot. Thorax: pronotal comb ♂ 26, ♀ 26. Abdomen: 2 bristles on each of tergum of sternum 1–7; antepygidial bristles ♂ 1, ♀ 3; ♂ apical appendage of median lamina or aedeagal apodeme curled into a spiral ribbon (the reason for the origin of the name), about 3 laps. The 9th tendon of sternum and the tendon of phallosome also curled 5 laps. The end of the process of clasper was enlarged, and the anterior and posterior horns were sharp. Movable process narrow and long, with 2 spiny bristles at the end, movable process with inverted bell-shaped base of posterior ventral process, its middle segment shorter than the end segment. ♀ Tergum ⅷ with upper lateral bristles growing radially towards the anterior superior, posterior superior and posterior inferior; has an anal pyramid with 6 bristles (Fig. S1).

### Mitogenome organization

**Figure 1.**
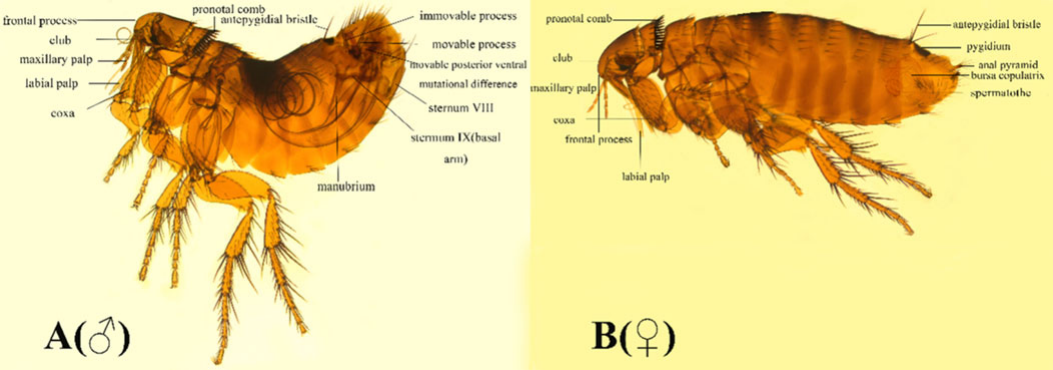
Organization of the *Amphalius spirataenius* mitogenome. tRNA genes were shown with the single-letter abbreviations of their corresponding amino acids. *Note*: The morphological figure of *Ochotona thibetana* from the volume 7 of *The Mammals of The World* (Wilson *et al*., 2017).

**Figure 2.**
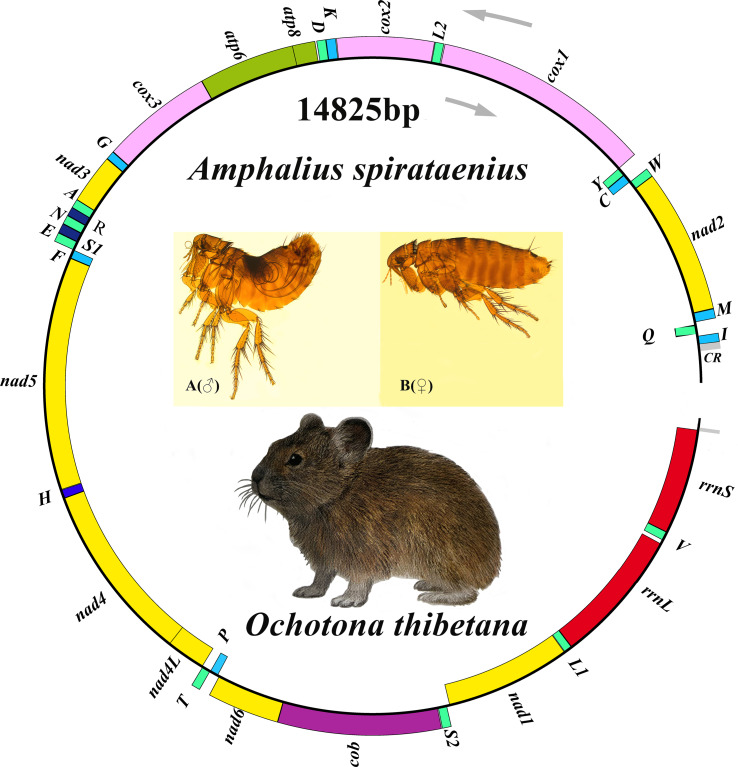
Skewness of 13 protein-coding genes of *Amphalius spirataenius*.

The *A. spirataenius* mitogenome (GenBank accession number: OR855715) was 14 825 bp, except for non-coding region (incomplete), with 37 genes of typical metazoan animals, including 13 PCGs (*cox1-3*, *nad1-6*, *nad4L*, *atp6*, *atp8*, *cob*), 22 tRNA genes and 2 rRNA genes (Table 1). Arrangement pattern of the *A. spirataenius* mitogenome retained that of hypothetical insect ancestors (Fig. 1). Base composition of the *A. spirataenius* mitogenome was A: 38.0%, T: 40.7%, G: 8.5%, C: 12.8%, with AT content of 78.7%. AT-skew was −0.034, GC-skew was −0.199. AT-skews of 13 PCGs were all negative, and GC-skews were all negative except for *nad1*, *nad4*, *nad4L* and *nad5* (Fig. 2). The length of *rrnL* was 1299 bp, and the length of *rrnS* was 779 bp. A total of 12 intergenic regions was 68 bp. The largest intergenic region was 19 bp, followed by 16 bp and the smallest was 1 bp. There were 10 overlaps (28 bp), the largest overlapping region was 7 bp, and the smallest was 1 bp. In the *A. spirataenius* mitogenome, 14 genes were encoded on the N strand (*trnQ*, *trnC*, *trnY*, *trnF*, *nad5*, *trnH*, *nad4*, *nad4L*, *trnP*, *nad1*, *trnL1*, *rrnL*, *trnV*, *rrnS*), and the remaining 23 genes were encoded on the J strand.

**Table 1.** Distribution of the *Amphalius spirataenius* mitogenome

					Nucleic acid (%)		Codon			
Name	Strand	Position	Length (bp)	Intergenic nucleotide	AT	GC	Start	Stop	Anti-codon	Amino acid size
PCGs	J		6858		76.5	23.6				
PCGs	N		4272		80.1	19.9				
1st codon position	J		2286		71.6	28.4				
1st codon position	N		1424		76.2	23.8				
2nd codon position	J		2286		66.5	33.5				
2nd codon position	N		1424		71.9	28.1				
3rd codon position	J		2286		91.4	8.6				
3rd codon position	N		1424		92.2	7.8				
*trnI*	J	1–63	63						GAU	
*trnQ*	N	65–133	69	1					UUG	
*trnM*	J	133–199	67	−1					CAU	
*nad2*	J	200–1208	1009		81.3	18.7	ATT	T		336
*trnW*	J	1209–1276	68						UCA	
*trnC*	N	1283–1343	61	6					GCA	
*trnY*	N	1344–1407	64						GUA	
*cox1*	J	1405–2940	1536	−3	71.1	28.8	ATC	TAA		512
*trnL*_2_*(taa)*	J	2945–3008	64	4					UAA	
*cox2*	J	3010–3690	681	1	75.4	24.5	ATG	TAA		227
*trnK*	J	3693–3762	70	2					CUU	
*trnD*	J	3762–3827	66	−1					GUC	
*atp8*	J	3828–4004	177		84.8	15.2	ATC	TAA		59
*atp6*	J	3998–4672	675	−7	77.4	22.5	ATG	TAA		225
*cox3*	J	4672–5454	783	−1	72.9	27.1	ATG	TAA		261
*trnG*	J	5455–5517	63						UCC	
*nad3*	J	5518–5868	351		79.5	20.5	ATT	TAG		117
*trnA*	J	5867–5930	64	−2					UGC	
*trnR*	J	5933–5995	63	2					UCG	
*trnN*	J	5993–6058	66	−3					GUU	
*trnS1(tct)*	J	6059–6126	68						UCU	
*trnE*	J	6127–6191	65						UUC	
*trnF*	N	6190–6253	64	−2					GAA	
*nad5*	N	6254–7967	1714		80.6	19.4	ATG	T		571
*trnH*	N	7969–8031	63	1					GUG	
*nad4*	N	8032–9367	1336		79.9	20.1	ATG	T		445
*nad4L*	N	9361–9654	294	−7	83.0	17.0	ATG	TAA		98
*trnT*	J	9657–9726	70	2					UGU	
*trnP*	N	9727–9789	63						UGG	
*nad6*	J	9801–10 307	507	11	86.6	13.4	ATT	TAA		169
*cob*	J	10 307–11 446	1140	−1	75.2	24.8	ATG	TAA		380
*trnS*_2_*(tga)*	J	11 449–11 512	64	2					UGA	
*nad1*	N	11 532–12 461	930	19	78.5	21.5	ATA	TAA		310
*trnL*_1_*(tag)*	N	12 478–12 539	62	16					UAG	
*rrnL*	N	12 540–13 838	1299							
*trnV*	N	13 839–13 906	68						UAC	
*rrnS*	N	13 907–14 685	779

### Protein-coding genes and codon usage bias

**Figure 3.**
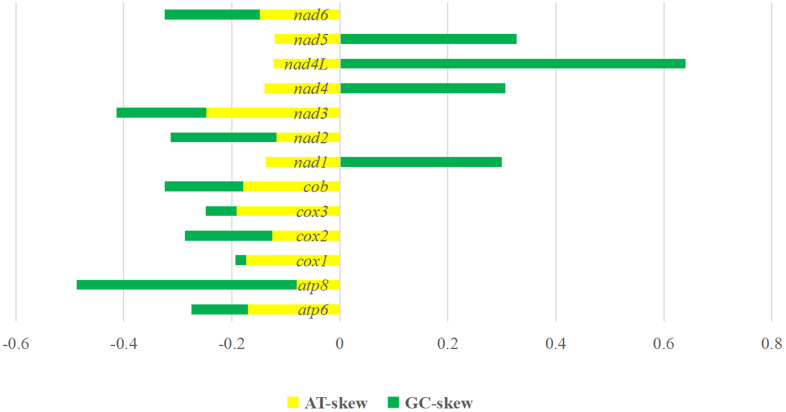
Relative synonymous codon usage (RSCU) of *Amphalius spirataenius*. The *Y*-axis represents the RSCU value, and the *X*-axis represents the codons corresponding to each amino acid.

The total length of 13 PCGs was 11 133 bp. The total length of PCGs on the N strand was 4274 bp, and the total length of PCGs on the J strand was 6859 bp. The longest PCG was *nad5* (1714 bp), and the shortest was *atp8* (177 bp). All PCGs began with the typical ATN as start codon, of which *nad2*, *nad3* and *nad6* use ATT as start codon, *cox1* and *atp8* use ATC as start codon, *nad1* uses ATA as start codon, and the remaining PCGs use ATG as start codon. Incomplete stop codon ‘T’ for *nad2*, *nad4* and *nad5*, while *nad3* use TAG as stop codon. The remaining 8 PCGs use TAA as complete stop codons (Table 1). The RSCU was calculated for the *A. spirataenius* mitogenome (Table 2, Fig. 3). The most frequently used codons were UUA (Leu) and UCU (Ser), while UCG (Ser) and ACG (Thr) were the least frequently used codons. Twenty-six codons of UUU, UUA, AUU, AUA, UAU, AAU, etc., were preference codons (RSCU > 1).

**Table 2.** Codon usage of protein-coding genes in the *Amphalius spirataenius* mitogenome

Amino acid	Codon	Number of uses	RSCU	Amino acid	Codon	Number of uses	RSCU
Phe	UUU	317	1.82	Thr	ACG	0	0
	UUC	32	0.18	Ala	GCU	78	2.33
Leu	UUA	479	4.96		GCC	6	0.18
	UUG	22	0.23		GCA	48	1.43
	CUU	49	0.51		GCG	2	0.06
	CUC	6	0.06	Tyr	UAU	170	1.84
	CUA	23	0.24		UAC	15	0.16
	CUG	1	0.01	His	CAU	59	1.74
Ile	AUU	366	1.88		CAC	9	0.26
	AUC	24	0.12	Gln	CAA	56	1.93
Met	AUA	267	1.77		CAG	2	0.07
	AUG	35	0.23	Asn	AAU	216	1.86
Val	GUU	56	1.67		AAC	16	0.14
	GUC	5	0.15	Lys	AAA	89	1.82
	GUA	70	2.09		AAG	9	0.18
	GUG	3	0.09	Asp	GAU	57	1.84
Ser	UCU	140	3.01		GAC	5	0.16
	UCC	13	0.28	Glu	GAA	66	1.71
	UCA	87	1.87		GAG	11	0.29
	UCG	0	0	Cys	UGU	36	1.85
	AGU	32	0.69		UGC	3	0.15
	AGC	2	0.04	Trp	UGA	88	1.83
	AGA	91	1.96		UGG	8	0.17
	AGG	7	0.15	Arg	CGU	13	0.96
Pro	CCU	80	2.6		CGC	2	0.15
	CCC	6	0.2		CGA	38	2.81
	CCA	33	1.07		CGG	1	0.07
	CCG	4	0.13	Gly	GGU	46	0.92
Thr	ACU	87	2.38		GGC	4	0.08
	ACC	6	0.16		GGA	102	2.03
	ACA	53	1.45		GGG	49	0.98

**Figure 4.**
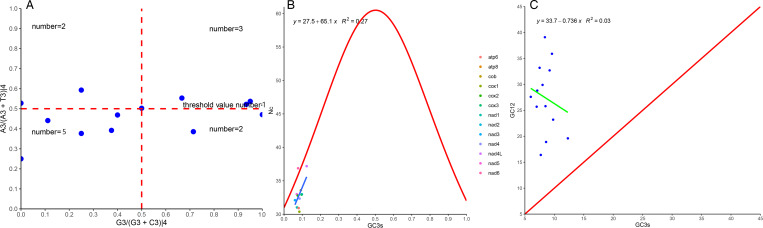
Analysis of 13 protein-coding genes of *Amphalius spirataenius*. (A) PR2; (B) ENC-plot; (C) neutral curve.

RSCU was mainly measured to know the relative probability of specific codon in the synonymous codon encoding the corresponding amino acid, which can intuitively reflect codon usage preference, but the reason for its preference was not clear. Parity rule 2 (PR2) (Sueoka, 1995), effective codon count (ENC-plot) (Wright, 1990) and neutrality curve (Patil *et al*., 2021) analyses of *A. spirataenius* were performed to evaluate factors that influenced evolutionary processes. PR2 indicated by qualitative analyses that mutation, selection and other factors together influence codon usage, and it was concluded that the third base of codons has T/C preference (Fig. 4A). In ENC-plot, except for the Nc values of *nad4L* (37.19) and *nad6* (36.87) > 35, the remaining PCGs were lower than 35 (Nc < 35) and most of them fall below standard curve (red) (Fig. 4B). Neutral curve indicated that regression coefficient was –0.736, and all values were above diagonal curve (red) (Fig. 4C).

The evolutionary rates of 13 PCGs were calculated and analysed using KAKS_Calculator2.016 software with *Drosophila Yakuba* (Clary and Wolstenholme, 1984) as the outgroup. Among 13 PCGs, *nad6* (Ka/Ks = 0.272) had the fastest evolutionary rate, followed by *atp8* (Ka/Ks = 0.167). Evolution rate of *cox1* (Ka/Ks = 0.024) was the slowest. The ratio rate (Ka/Ks) of 13 PCGs was lower than 1 (Fig. S2). The proportion of conserved sites among 13 PCGs was the highest for *cox1* (0.809), lowest for *atp8* (0.598) and second for *nad6* (0.636). The percentage of variant sites for *nad2* (0.329) was the highest, second lowest for *nad6* (0.318) and lowest for *cox1* (0.190). The nucleotide diversity of *atp8* gene was 0.345, which was the most diverse nucleotide among all PCGs genes, followed by *nad2* (Pi = 0.337) and *nad6* (Pi = 0.333) (Table S2, Fig. S2).

### tRNA and rRNA gene analysis

Among 22 tRNA genes, the longest was 70 bp (*trnK* and *trnT*), the shortest was 62 bp (*trnL_1 (tga)_*), and the average length was 65.2 ± 2.7 bp. The anticodons of *trnK* (CUU) and *trnS_1_* (UCU) in *A. spirataenius* were different from those of some other arthropod mitochondrial *trnK* (UUU) and *trnS_1_* (GCU) (Sun *et al*., 2022; Yang *et al*., 2023; Yuan *et al*., 2023). During tRNA gene folding, in addition to the typical Watson–Crick (A–U, G–C) pairing, there were 19 mismatch base pairs, of which there were 15 G–U mismatches, 2 U–U mismatches, 1 C–A mismatch and 1 C–U mismatch. The remaining 21 tRNA genes formed the typical cloverleaf structures, except for *trnS_1_* which lacked the D-arm (Fig. S3). The lengths of 2 rRNA genes were 1299 bp (*rrnL*) and 779 bp (*rrnS*) respectively, where AT/GC content of *rrnL* gene was 81.9%/12.3%, while that of *rrnS* gene was 80.4%/12.7%, both of which were encoded on the N strand (Table 1).

### Phylogenetic analysis

**Figure 5.**
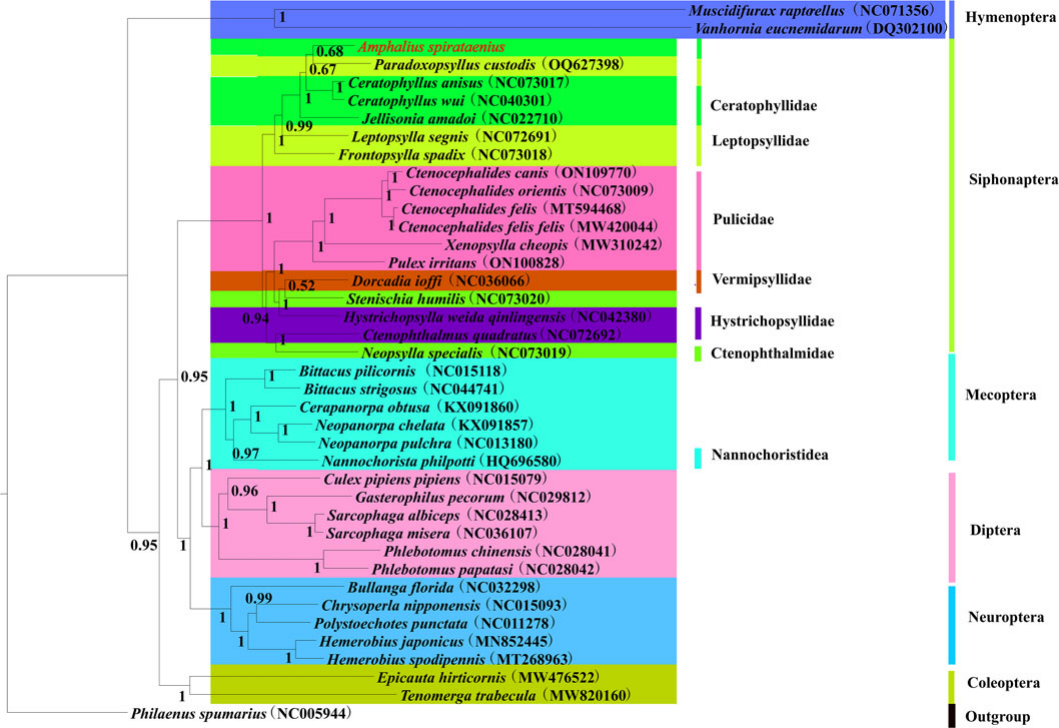
Phylogenetic tree of 40 insect species was constructed using Bayesian methods with *Philaenus spumarius* as the outgroup and node values as posterior probability values (PP). *Amphalius spirataenius* was labelled in red.

**Figure 6.**
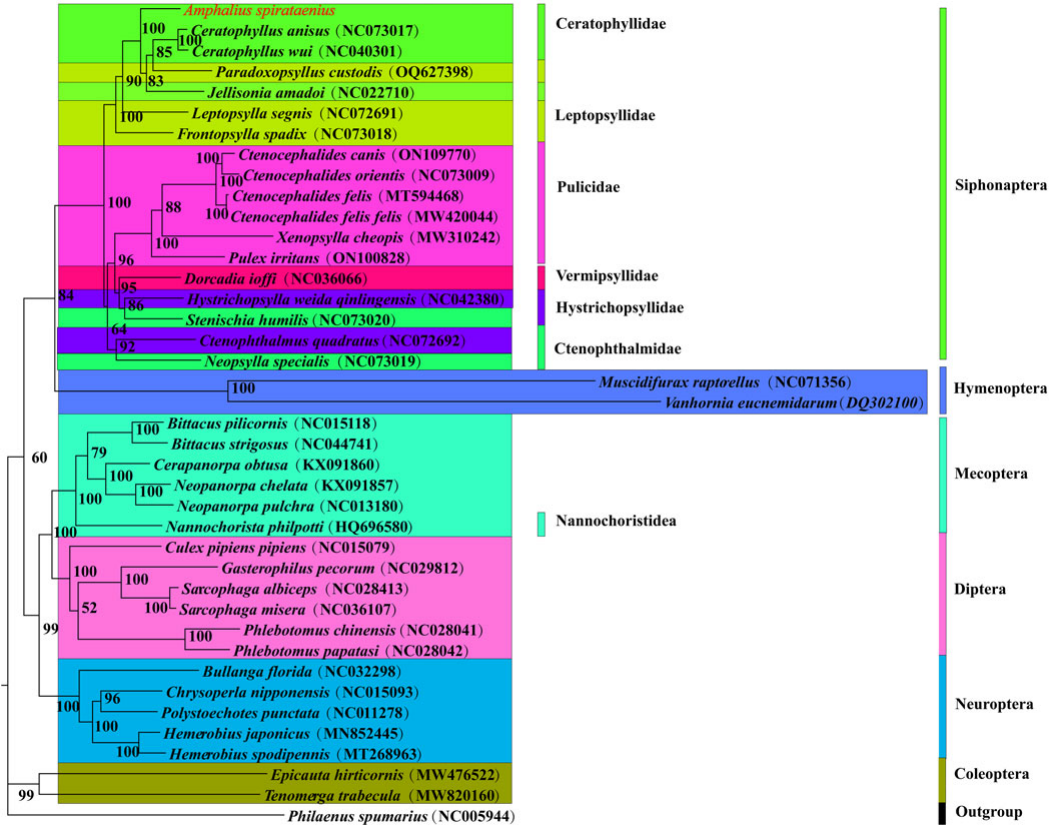
Phylogenetic tree of 40 insect species was constructed by maximum likelihood method with *Philaenus spumarius* as an outgroup and node values as bootstrap values (BS). *Amphalius spirataenius* was labelled in red.

Concatenated nucleotide sequences of 13 PCGs and 2 rRNA genes (13 PCGs + 2 rRNAs) from the mitogenomes of 40 insect species (18 flea species of Siphonaptera) were analysed using BI and ML method to construct a phylogenetic tree with *P. spumarius* (NC005944) as outgroup. The 2 phylogenetic tree topologies are slightly different, but both have high node support (posterior probabilities >95%, bootstrap values >70%) (Figs 5 and 6). The phylogenetic tree the hypothesis supported that the order Siphonaptera is monophyly and formed the major clade with 3 branches, with the Bayesian posterior probability (Bpp = 1) and the Ultrafast bootstrap approximation value (UFBoot = 100%) in the BI and ML analyses, respectively.

## Discussion

The *A. spirataenius* mitogenome was reported for the first time in this study. The 14 825 bp long mitogenome has the typical metazoan of 37 genes with insect ancestral genome arrangement patterns (Clary and Wolstenholme, 1984). To date, the mitogenomes of 18 flea species have been sequenced in the world and their AT/GC contents are shown in Table S1. The AT content of fleas is as high as 76.7–83.2%, which is much higher than that of parasitic lice (*Polyplax asiatica* AT = 58%, *Polyplax spinulosa* AT = 61%) (Zhang and Dong, 2020) and the 2 orders Mecoptera and Diptera (Song *et al*., 2016*a*, 2016*b*). High AT content may be the main reason for incomplete sequencing or sequencing failure of control regions. AT-skew indicated that the remaining 17 flea species had negative AT-skew except for *Leptopsylla segnis* (AT-skew = 0.024) and *Neopsylla specialis* (AT-skew = 0), while GC-skew was positive for the remaining 17 flea species except for *Leptopsylla segnis* (GC-skew = 0.248), which might be resulted from directional evolutionary pressure and asymmetric replication. It might be also related to living environment and parasitic life history because only adult fleas parasitize on host. Negative GC-skew in the J-strand and positive GC-skew in the N-strand of *A. spirataenius* were consistent with the mitogenome of most metazoan (Dermauw *et al*., 2009).

There are 12 intergenic spacers in the *A. spirataenius* mitogenome, with the largest intergenic spacer (19 bp) located between *trnS_2_* and *nad1* (Table 1). Intergenic spacer might be transcription termination signal site of transcription process (Cameron and Whiting, 2008; Yang *et al*., 2023). There were 10 gene overlaps, with the largest overlap region of 7 bp (*atp6* and *atp8*; *nad4* and *nad4L*) and the overlap region between *atp6* and *atp8* is common in arthropod mitogenomes (Ge *et al*., 2022). Some researchers believed that gene intergenic and overlap regions of mitogenome might be beneficial for mitochondrial structural stability (Song *et al*., 2016*a*, 2016*b*).

The 13 PCGs of *A. spirataenius* had typical ATN as the start codon. In fact, the start codons of 13 PCGs in some metazoans are not entirely typical ATNs. For example, *atp8* of *Eulaelaps huzhuensis* used GTG as the start codon (Yang *et al*., 2023). Most PCGs of Mecoptera and Diptera used TCG as the start codon (Clary and Wolstenholme, 1984; Beckenbach, 2011), and the *cox1* of some insects used GCA as the start codon (Cameron, 2015). It was often the case that *cox1* of insects had atypical start codons, most likely due to the 1 bp deletion that caused the TCG frameshift (Cameron, 2015). *Nad2*, *nad4* and *nad5* of *A. spirataenius* had incomplete stop codon ‘T’, while *nad3* used TAG as stop codon. The remaining 8 PCGs used the typical TAN as stop codon. PCGs of many arthropods use incomplete stop codons (Liu *et al*., 2022). These incomplete stop codons might be transcribed by polyadenylation to obtain the complete stop codon TAA (Huang *et al*., 2022).

We calculated the RSCU for 13 PCGs of *A. spirataenius* for codon usage preference assessment (Fig. 3). Twenty-six codons of UUU, UUA, AUU, AUA, UAU, AAU, etc., were preference codons (RSCU > 1), RSCU > 1 indicated relatively high codon usage frequency (Liu *et al*., 2016; Yang *et al*., 2023). Most codons ending with A/U bases were frequently used, while codons ending with G/C were rarely used, or even in some cases not used in the *A. spirataenius* mitogenome, which was consistent with the RSCU of other metazoan (Hao *et al*., 2017). There were some exceptions, such as Diptera's preference for codons ending in G/C (Vicario *et al*., 2007; Behura and Severson, 2011). Generally, codon usage patterns were more similar between closely related species. In order to evaluate factors that influenced evolutionary processes, PR2 (Fig. 4A), ENC-plot (Fig. 4B) and neutral curve analyses (Fig. 4C) of *A. spirataenius* were performed. PR2, ENC-plot and neutrality curve analyses indicated that the influence of codon usage preference of *A. spirataenius* might be mainly resulted from selection pressure. Additionally, base composition (Arhondakis *et al*., 2004), overall expression level of gene (Hiraoka *et al*., 2009), nature of amino acids (aromatic and hydrophobic) (Knight *et al*., 2001) and codon context (Jia and Higgs, 2008) might also be influenced by codon usage preference.

The evolutionary rates of 13 PCGs were calculated and analysed using KAKS_Calculator2.016 software. Ka/Ks < 1 of 13 PCGs of *A. spirataenius* indicated that PCGs were subject to negative or purifying selection, and slow evolution. Combined with Ka/Ks, conserved sites, variable sites and nucleotide diversity analysis, it was found that *nad6* had the fastest evolutionary rate, followed by *atp8* and *nad2* in the *A. spirataenius* mitogenome. *Cox1* had the slowest evolution rate and is suitable for phylogenetic analysis or species classification. In 2023, Jakovlić *et al.* proposed that the evolutionary rates of bilaterally symmetrical animals are in descending order: internal parasites > weakly motile ectoparasites > weakly motile and free-living animals > parasitoid lineages > strongly motile ectoparasites > micropredators and strongly motile free-living animal (Jakovlić *et al*., 2023). Fleas are strongly motile ectoparasites that may have been subjected to stronger selective pressures and so have evolved at lower rate, whereas some ectoparasites may have undergone accelerated evolution due to slack selective pressures, where some mutations had no effect on its life activities. The reason for the slow evolutionary rate of fleas may also be related to their life history, parasitism and metabolic rate in the body. Species evolution was a complex and variable process, and apart from the above possible reasons, there are some other factors such as generation time, replication and repair machinery, directional selection driven by host parasite arms race, etc. (Dawkins and Krebs, 1979; Haraguchi and Sasaki, 1996; Jakovlić *et al*., 2023).

The average length of 22 tRNA genes of *A. spirataenius* was 65.2 ± 2.7 bp, which was longer than that of Parasitiformes (62.0 ± 1.3 bp) (Yuan *et al*., 2010). The anticodon of *trnK* (CUU) was different from that of some other arthropod *trnK* (UUU) (Sun *et al*., 2022; Yuan *et al*., 2023); *trnS_1_* used UCU as an anticodon, whereas *trnS_1_* of most arthropods used GCU as an anticodon (Yang *et al*., 2023). The secondary structures of 22 tRNA genes of *A. spirataenius* are shown in Fig. S3; of which 21 genes had typical cloverleaf structure except for *trnS_1_* which lacked the D-arm. Generally, *trnS_1_* which lacked the D-arm was prevalent in metazoan (Wang *et al*., 2012). The tRNA secondary structure might be related to species evolution (Watanabe *et al*., 1994). The tRNA secondary structure of *A. spirataenius* had 19 mismatch base pairs, of which there were 15 G–U mismatches, 2 U–U mismatches, 1 C–A mismatch and 1 C–U mismatch. G–U mismatches were favourable to the maintenance of the tRNA secondary structure (He and Dong, 2023) and G–U mismatches mostly occurred at turn point of the tRNA secondary structure.

The phylogenetic tree demonstrated that *A. spirataenius*, *Ceratophyllus anisus*, *Ceratophyllus wui*, *Paradoxopsvllus custodis* and *Jellisonia amadoi* were sister groups (PP = 1, BS = 100) and species of the same family or genus with well-defined taxonomic status were clustered together. It was consistent with the traditional morphological classifications. Ceratophyllidae and Leptopsyllidae were clustered into 1 major clade, while Pulicidae, Ctenophthalmidea, Hystrichopsylloidea and Vermipsyllidae were clustered into the other major clade. Ceratophyllidae formed sister group with the other 5 families. The families Ceratophyllida, Leptopsyllidae, Ctenophthalmidea and Hystrichopsylloidea were paraphyletic. Interestingly, *Dorcadia ioffi* were clustered with *Hystrichopsylla weida qinlingensis* with high support (PP = 1.0, BS = 95), which was somewhat inconsistent with the view of Wu (2007). The superfamily Vermipsylloidea and Hystrichopsylloidea were separated from Ceratophylloidea forming 2 independent families respectively. Interestingly, 3 superfamilies had obvious differences in morphology, but belong to the same clade in the phylogenetic tree. Phylogenetic relationship will be further explored by enlarged sample size in the future.

The phylogenetic tree strongly supported that the orders Siphonaptera and Mecoptera were monophyly. It was accordant to previous studies (Misof *et al*., 2014; Meusemann *et al*., 2020). The order Hymenoptera was the earliest clade. In recent years, some researchers suggested that the order Siphonaptera formed a sister group with the family Nannochoristidea, and the order Siphonaptera was treated as a member (the infraorder Siphonaptera) of the order Mecoptera (Tihelka *et al*., 2020). However, the family Nannochoristidea and the order Siphonaptera were a separate clade, respectively, in the phylogenetic tree. The order Siphonaptera was closely related to the orders Neuroptera, Diptera and Mecoptera and formed a sister group (PP = 0.95, BS = 60) (Figs 5 and 6). These results have provided new insights into the phylogenetic position of the order Siphonaptera within holometabolous insects. However, in current study, all lineages of fleas were not included in the analyses. Therefore, further study involving more mitogenomes of all flea families in the order Siphonaptera was needed to reassess phylogenetic relationship of the order Siphonaptera within holometabolous insects and obtain more reliable results.

## Conclusion

Organization and evolution of the *A. spirataenius* mitogenome were reported for the first time in this study. It provided unique insights into the phylogenetic and taxonomic status of the order Siphonaptera, which were monophyletic and belong to order level rather than infraorder level. The *A. spirataenius* mitogenome provides new molecular data for phylogeny and taxonomic level of the order Siphonaptera. To obtain a more reliable phylogenetic tree, we still need to collect more representative species of the order Siphonaptera and to sequence the mitogenomes of more species, and to study evolutionary mechanisms of the order Siphonaptera in depth.

## Supporting information

Pu et al. supplementary material 1 Pu et al. supplementary material

Pu et al. supplementary material 2 Pu et al. supplementary material

## Data Availability

All data generated or used during the study appear in our manuscript.

## Supplementary material

The supplementary material for this article can be found at https://doi.org/10.1017/S0031182024000635.
