# The first mitogenome of the genus Amphalius (Siphonaptera: Ceratophyllidae) and its phylogenetic implications – CORRIGENDUM

**DOI:** 10.1017/S0031182024001604

**Published:** 2024-10

**Authors:** Ju Pu, Xiaoxia Lin, Wenge Dong

The author regrets the inclusion of the below errors in the above article. These errors concern the numbering of several figures and supplementary figures.

The correct list is as follows:

**Figures**

**Figure 1.**
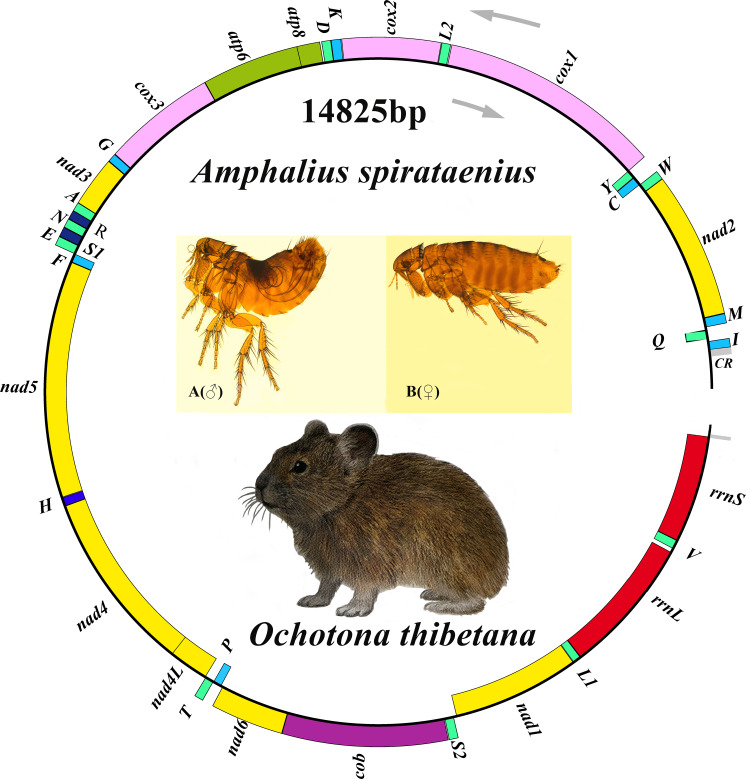
Organization of the *Amphalius spirataenius* mitogenome. tRNA genes were shown with the single-letter abbreviations of their corresponding amino acids. (note: The morphological figure of *Ochotona thibetana* from the volume 7 of *The Mammals of The World* (Wilson *et al.*, 2017))

**Figure 2.**
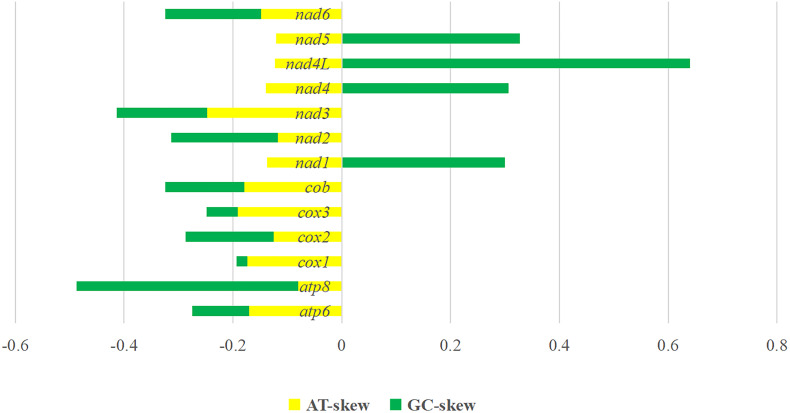
Skewness of 13 protein-coding genes of *Amphalius spirataenius*

**Figure 3.**
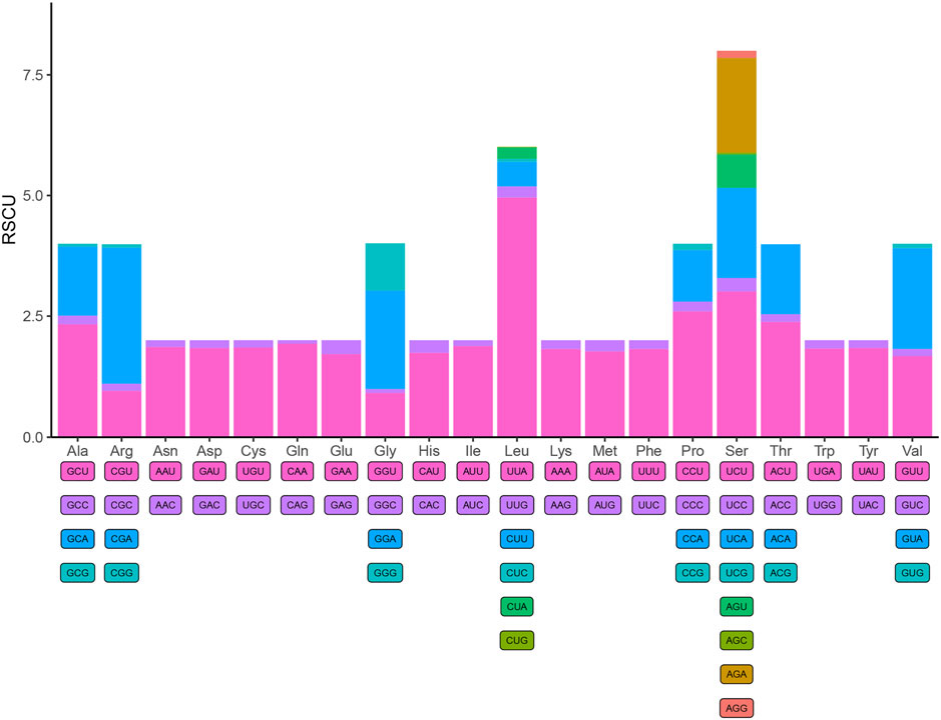
Relative synonymous codon usage (RSCU) of *Amphalius spirataenius*. The Y-axis represents the RSCU value, and the X-axis represents the codons corresponding to each amino acid

**Figure 4.**
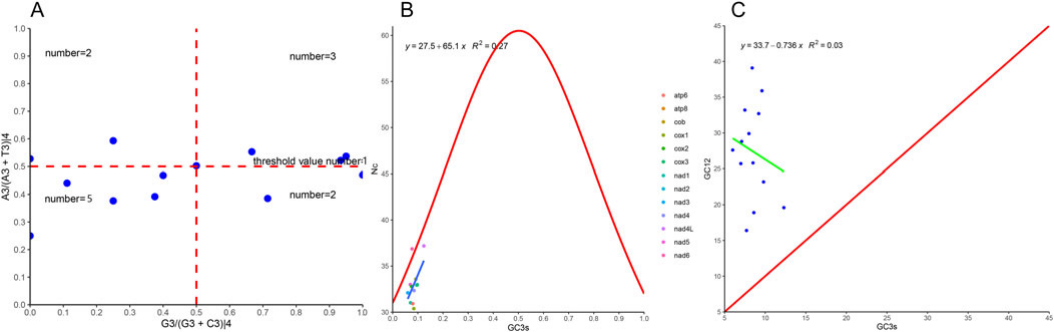
Analysis of 13 protein-coding genes of *Amphalius spirataenius*. A PR2; B ENC-plot; C Neutral curve

**Figure 5.**
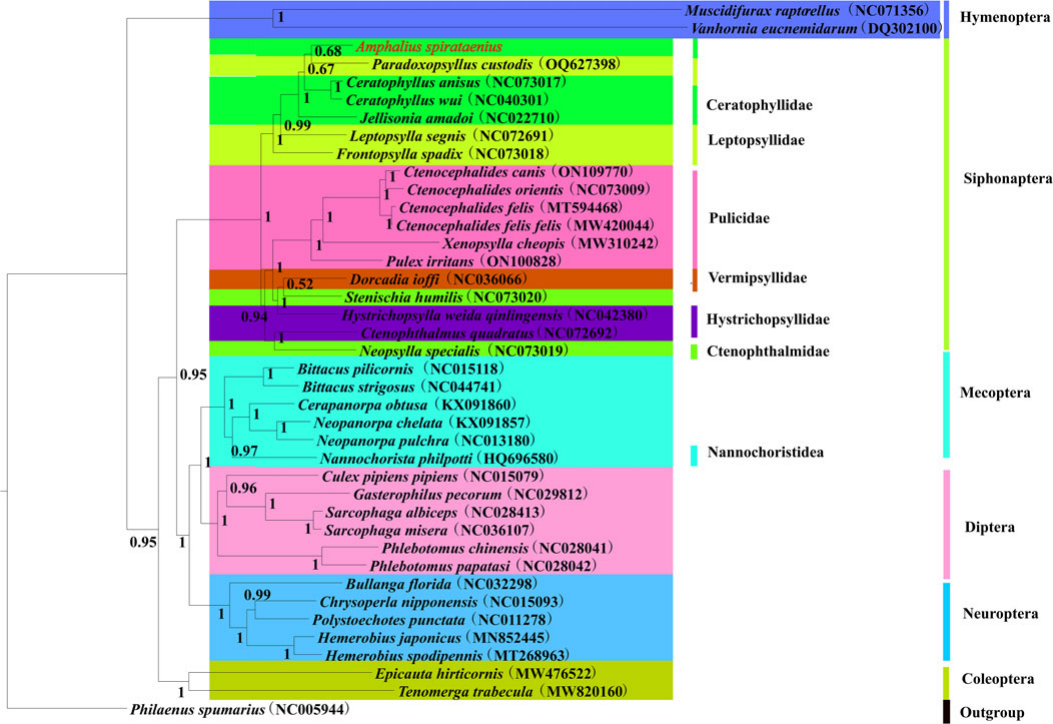
Phylogenetic tree of 40 insect species was constructed using Bayesian methods with *Philaenus spumarius* as the outgroup and node values as posterior probability values (PP). *Amphalius spirataenius* was labelled in red

**Figure 6.**
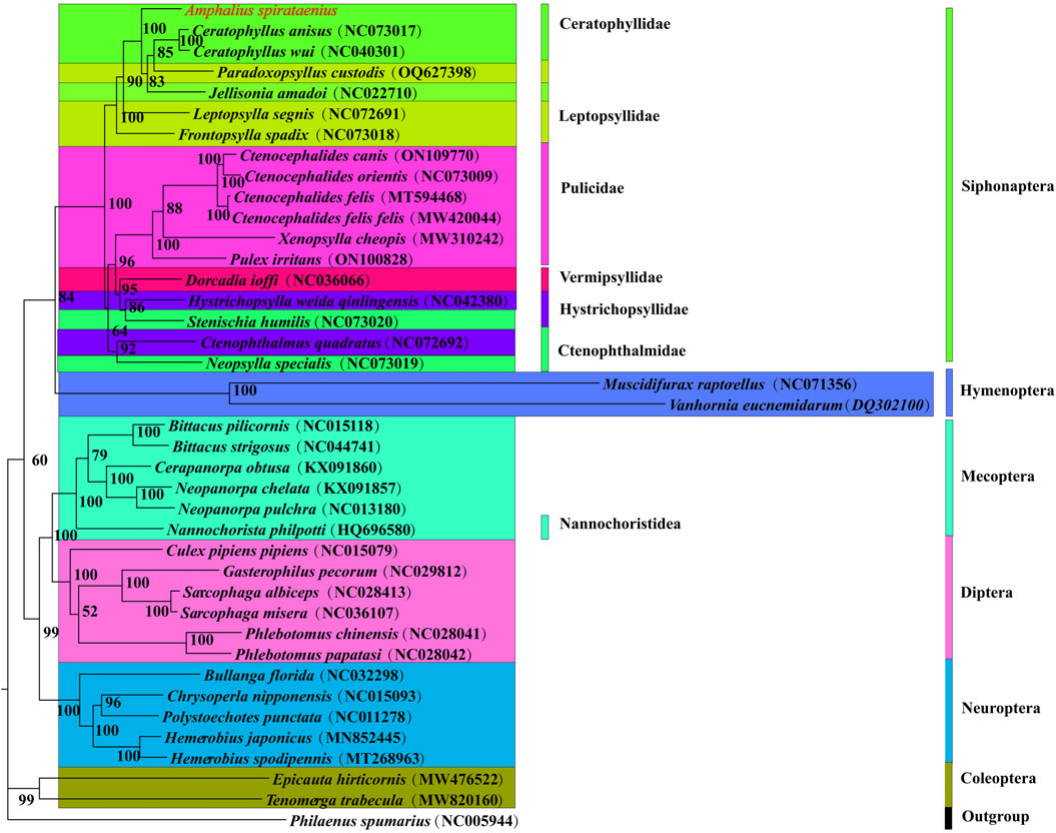
Phylogenetic tree of 40 insect species was constructed by Maximum likelihood method with *Philaenus spumarius* as an outgroup and node values as bootstrap values (BS). *Amphalius spirataenius* was labelled in red

**Supplementary Figures**

The author apologises for these errors and wishes to correct them through this notice.

## References

[ref1] Pu J, Lin X, Dong W. The first mitogenome of the genus Amphalius (Siphonaptera: Ceratophyllidae) and its phylogenetic implications. Parasitology. Published online 2024:1–11. doi:10.1017/S0031182024000635 PMC11894015 39623585

