# Supplementary figures and images for: The first mitogenome of the genus Amphalius (Siphonaptera: Ceratophyllidae) and its phylogenetic implications
Source: Parasitology. 2024 Dec 3;151(10):1085–95. doi: 10.1017/S0031182024000635 (PMC11894015; doi:10.1017/S0031182024000635)

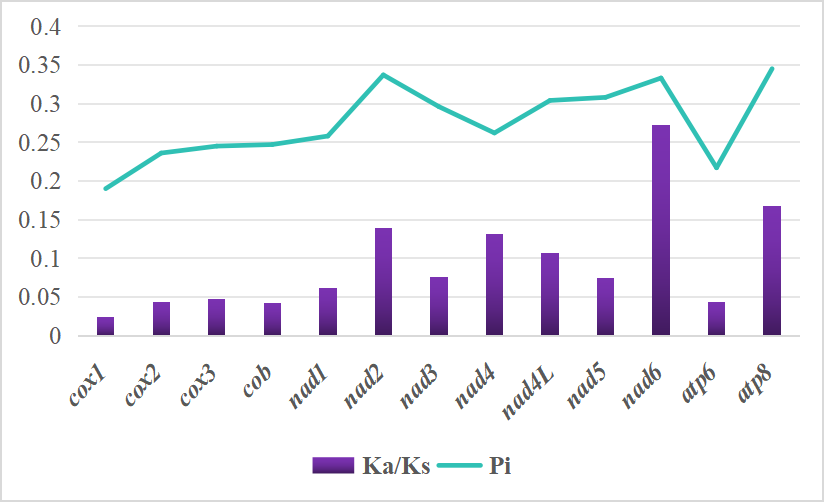

Supplement: Pu et al. supplementary material 1 — Pu et al. supplementary material [file S0031182024000635sup001.tif]
