# Supplementary material for: The first mitogenome of the genus Amphalius (Siphonaptera: Ceratophyllidae) and its phylogenetic implications
Source: Parasitology. 2024 Dec 3;151(10):1085–95. doi: 10.1017/S0031182024000635 (PMC11894015; doi:10.1017/S0031182024000635)
Supplement: Pu et al. supplementary material 2 — Pu et al. supplementary material [file S0031182024000635sup002.docx]

**Table S1** Nucleotide composition of the order Siphonaptera mitogenome

| Name | accession number | AT% | AT-skew | GC-skew |
| --- | --- | --- | --- | --- |
| *Amphalius spirataenius* | OR855715 | 78.7 | -0.034 | -0.199 |
| *Jellisonia amadoi* | NC022710 | 79.2 | -0.02 | -0.26 |
| *Stenischia humilis* | NC073020 | 78 | -0.011 | -0.238 |
| *Ceratophyllus anisus* | NC073017 | 78.5 | -0.022 | -0.231 |
| *Ctenocephalides orientis* | NC073009 | 83.2 | -0.051 | -0.258 |
| *Leptopsylla segnis* | NC072691 | 78.9 | 0.024 | 0.248 |
| *Ctenocephalides felis* | MT594468 | 83.1 | -0.044 | -0.23 |
| *Ctenocephalides felis felis* | MW420044 | 82.9 | -0.044 | -0.237 |
| *Ctenocephalides canis* | ON109770 | 78.5 | -0.017 | -0.182 |
| *Pulex irritans* | ON100828 | 80 | -0.027 | -0.146 |
| *Paradoxopsyllus custodis* | OQ627398 | 76.8 | -0.008 | -0.259 |
| *Hystrichopsylla weida qinlingensis* | NC042380 | 80.6 | -0.03 | -0.221 |
| *Neopsylla specialis* | NC073019 | 77.2 | 0 | -0.251 |
| *Dorcadia ioffi* | NC036066 | 80.7 | -0.006 | -0.198 |
| *Xenopsylla cheopis* | MW310242 | 82.8 | -0.01 | -0.221 |
| *Frontopsylla spadix* | NC073018 | 78.8 | -0.036 | -0.214 |
| *Ctenophthalmus quadratus* | NC072692 | 79.5 | -0.014 | -0.226 |
| *Ceratophyllus wui* | NC040301 | 76.7 | -0.017 | -0.184 |

**Table S2.** Conserved sites, varianble sites, Ka/Ks and nucleotide diversity of 13 PCGs of *Amphalius spirataenius*

| Gene | C | V | Ka/Ks | Pi |
| --- | --- | --- | --- | --- |
| *cox1* | 1243 | 293 | 0.024 | 0.190 |
| *cox2* | 520 | 161 | 0.043 | 0.236 |
| *cox3* | 591 | 192 | 0.047 | 0.245 |
| *cob* | 856 | 281 | 0.042 | 0.247 |
| *nad1* | 690 | 240 | 0.062 | 0.258 |
| *nad2* | 666 | 339 | 0.139 | 0.337 |
| *nad3* | 247 | 104 | 0.076 | 0.296 |
| *nad4* | 984 | 351 | 0.131 | 0.262 |
| *nad4L* | 194 | 85 | 0.107 | 0.304 |
| *nad5* | 1177 | 524 | 0.074 | 0.308 |
| *nad6* | 336 | 168 | 0.272 | 0.333 |
| *atp6* | 528 | 147 | 0.043 | 0.217 |
| *atp8* | 106 | 56 | 0.167 | 0.345 |

Note: C: conserved sites, V: variable sites, Pi: Nucleotide diversity
